# Remdesivir: Effectiveness and Safety in Hospitalized Patients with COVID-19 (ReEs-COVID-19)—Analysis of Data from Daily Practice

**DOI:** 10.3390/microorganisms11081998

**Published:** 2023-08-03

**Authors:** Nikos Pantazis, Evmorfia Pechlivanidou, Anastasia Antoniadou, Karolina Akinosoglou, Ioannis Kalomenidis, Garyfallia Poulakou, Haralampos Milionis, Periklis Panagopoulos, Markos Marangos, Ioannis Katsarolis, Pinelopi Kazakou, Vasiliki Dimakopoulou, Anna-Louiza Chaliasou, Vasiliki Rapti, Eirini Christaki, Angelos Liontos, Vasileios Petrakis, Georgios Schinas, Dimitrios Biros, Maria-Christina Rimpa, Giota Touloumi

**Affiliations:** 1 Department of Hygiene, Epidemiology and Medical Statistics, Medical School, National & Kapodistrian University of Athens, 11527 Athens, Greece; 2 4th Department of Internal Medicine, Attikon University General Hospital, Medical School, National & Kapodistrian University of Athens, 12461 Athens, Greece; 3 Department of Internal Medicine, University General Hospital of Patras, Department of Medicine, University of Patras, 26504 Rio, Greece; 4 1st Department of Critical Care & Pulmonary Service, Evangelismos General Hospital, Medical School, National & Kapodistrian University of Athens, 10676 Athens, Greece; 5 COVID-19 Unit, Evangelismos General Hospital, 10676 Athens, Greece; 6 3rd Department of Internal Medicine, Athens Hospital for Diseases of the Chest “Sotiria”, Medical School, National & Kapodistrian University of Athens, 11527 Athens, Greece; 7 Department of Internal Medicine, University General Hospital of Ioannina, Faculty of Medicine, School of Health Sciences, University of Ioannina, 45500 Ioannina, Greecedimitrisbiros@gmail.com (D.B.); 8 Department of Internal Medicine, University General Hospital of Alexandroupolis, Department of Medicine, Democritus University of Thrace, 68100 Alexandroupolis, Greece; 9 Medical Affairs, Gilead Sciences Hellas and Cyprus, 17564 Paleo Faliro, Greece

**Keywords:** COVID-19, remdesivir, hospitalized patients, effectiveness, safety

## Abstract

Remdesivir was the first antiviral approved for treating COVID-19. We investigated its patterns of use, effectiveness and safety in clinical practice in Greece. This is a retrospective observational study of hospitalized adults who received remdesivir for COVID-19 in September 2020–February 2021. The main endpoints were the time to recovery (hospital discharge within 30 days from admission) and safety. The “early” (remdesivir initiation within 24 h since hospitalization) and “deferred” (remdesivir initiation later on) groups were compared. One thousand and four patients (60.6% male, mean age 61 years, 74.3% with severe disease, 70.9% with ≥1 comorbidities) were included, and 75.9% of them were on a 5-day regimen, and 86.8% were in the early group. Among those with a baseline mild/moderate disease, the median (95% CI) time to recovery was 8 (7–9) and 12 (11–14) days for the early and deferred groups, respectively (*p* < 0.001). The corresponding estimates for those with a severe disease were 10 (9–10) and 13 (11–15) days, respectively (*p* = 0.028). After remdesivir initiation, increased serum transaminases and an acute kidney injury were observed in 6.9% and 2.1%, respectively. Nine (0.9%) patients discontinued the treatment due to adverse events. The effectiveness of remdesivir was increased when it was taken within 24 h since admission regardless of the disease severity. Remdesivir’s safety profile is similar to that described in clinical trials and other real-world cohorts.

## 1. Introduction

COVID-19 was characterized as a pandemic on 12 March 2020 by the World Health Organization (WHO). Since that time, over 760 million people have been diagnosed with COVID-19 worldwide, and 6.9 million of them have died [1]. An age > 65 years, having a cardiovascular disease, diabetes mellitus, chronic obstructive pulmonary disease (COPD), obesity, and smoking have been identified as major risk factors for severe clinical outcomes of COVID-19 [2,3,4,5].

Remdesivir was the first antiviral used for the treatment of COVID-19 that was conditionally approved on 3 July 2020 by the European Medicines Agency (EMA) and on 22 October 2020 by the US Food and Drug Administration (FDA) [6,7,8]. Since 8 August 2022, remdesivir has gained full and unconditional recommendations by the EMA.

The approval of and recommendations for remdesivir by major scientific societies and clinical bodies were mainly based on the results of the ACTT-1 study [9], but also on two other clinical trials (GS-US-540-5773; GS-US-540-5774) [10,11]. In ACTT-1, the median time to recovery was 5 days faster in the remdesivir group compared to that of the placebo group [9]. In the final WHO’s Solidarity trial report, remdesivir was found to have a small, but still significant, impact on mortality and progression to ventilation for COVID-19 patients [12]. Nevertheless, the results from the two major studies (ACTT-1 and Solidarity) showed that remdesivir’s efficacy can differ depending on the time of administration, with the recovery rates being higher when given to patients within 10 days of symptom onset.

The objective of the ReEs-COVID-19 observational study was to describe patterns of remdesivir use, effectiveness, and safety in the treatment of hospitalized patients with COVID-19 in Greece and to investigate factors that could potentially affect remdesivir’s effectiveness. We hypothesized that the early (within 24 h since hospitalization) initiation of remdesivir is associated with faster recovery among patients than later initiation is and that the safety profile of the drug is similar to that observed in previous international trials.

## 2. Materials and Methods

### 2.1. Study Design and Eligibility Criteria

ReEs-COVID-19 is a retrospective observational cohort study of patients who received remdesivir as part of their care for a documented SARS-CoV-2 infection for at least 24 h. Adults (≥18 years old) with a documented positive PCR test result for SARS-CoV-2 who were hospitalized in one of the 6 collaborating clinics between 1 September 2020 and 28 February 2021 were included in the study. This time frame corresponds to the period during which (a) remdesivir was formally included as an approved therapeutic option in the National Treatment Algorithm and was readily available in Greek hospitals, and (b) the 2nd wave of the pandemic was in effect, just before the start of the mass vaccination campaign in the country. Patients who had to be admitted to an intensive care unit (ICU) on the first day of their hospitalization and patients who participated in clinical trials for other COVID-19 investigational treatments were excluded.

Data were collected retrospectively from patients’ files and hospitals’ records. The information gathered included baseline demographic and social characteristics, clinical status at the beginning of hospitalization, disease history, and known risk factors for disease progression. In addition, information about the course and outcome of the hospitalization, along with any observation of elevated serum transaminases and acute kidney injury up to 30 days after admission, was recorded.

### 2.2. Endpoints

The primary endpoint was the time from hospital admission to recovery. Recovery was defined as being discharge from hospital (with or without limitation of activities) or not requiring supplemental oxygen and no longer requiring ongoing medical care (i.e., reaching stages 1–3 of the 8-point ordinal scale of clinical status [9]) within 30 days after hospital admission. The time to recovery, as defined above, and the time to discharge from the COVID-19 clinics coincide for the studied period in all Greek hospitals. Given the burden of the health system in Greece during that period, the extension of a hospital stay in a COVID-19 clinic for managing other underlying conditions was extremely unlikely. In cases where the patients remained in the hospital for the treatment of other pre-existing conditions, they were transferred to other (non-COVID-19) clinics, and they were considered to have recovered at the day of discharge from the COVID-19 clinic. Additionally, as a secondary endpoint, the clinical status as defined using the 8-point ordinal scale was evaluated at 15 and 30 days after admission.

The other secondary endpoints included the description of patterns of remdesivir use through the time between admission and 1st dose and the total duration of administration; patients were classified into a) the early group: those who were given remdesivir the same day the patient was hospitalized or the next morning in cases where the patient entered the hospital late at night (i.e., <24 h since hospital admission); b) deferred group: those for whom remdesivir was initiated after 24 h post-hospital admission.

The time since hospital admission instead of the time since symptoms’ onset was used for the definition of the two groups due to the fact that the date of symptoms’ onset was self-reported, and thus, subject to error. Additionally, this choice ensured that the classification of “early” and “deferred” remdesivir initiators was based on real-life clinical practice using a well-defined time origin (i.e., hospital admission) and in line with the guidelines regarding remdesivir use.

The duration of remdesivir administration was classified as (a) <5 days, (b) 5 days, (c) 6–10 days, or (d) >10 days. It should be noted that during the study period, the national guidelines suggested a standard remdesivir scheme of 200 mg intravenous on day 1, followed by 100 mg for the subsequent 4 days for patients in need of supplemental oxygen, and recommended that the window period for remdesivir initiation was within the first 7 days since receiving a positive PCR test and/or the symptoms started [13].

Safety evaluation was also included as a primary endpoint for the analysis. Serum transaminase levels were classified in two stages based on alanine aminotransferase (ALT) and aspartate aminotransferase (AST) thresholds: (i) stage 1: ALT and/or AST between 3 and 5 × the upper limit of normal levels (ULN); (ii) stage 2: ALT and/or AST above 5 × ULN. To study renal function, the three-stage classification proposed by the Acute Kidney Injury (AKI) working group (KDIGO) was used [14,15]. The discontinuation of remdesivir was also recorded, as were ICU admissions and deaths for the 30-day follow-up period after remdesivir initiation.

**Figure 1:**
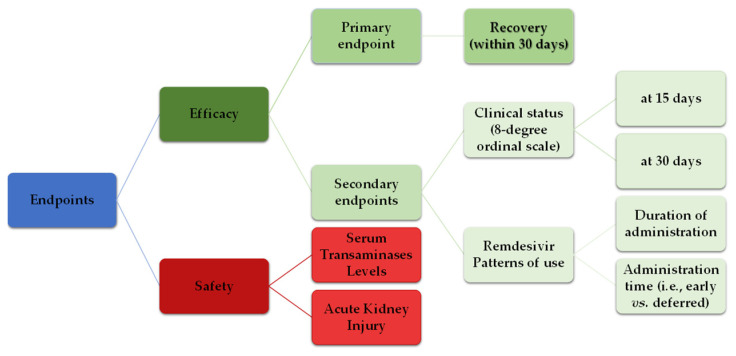
Study endpoints.

All study endpoints are presented graphically in Figure 1.

### 2.3. Ethics

The Bioethics and Deontology Committee of the Medical School of the National and Kapodistrian University of Athens (protocol number 505/19-04-2021) along with the Scientific Council of each hospital participating in ReEs-COVID-19 approved the study. The data were recorded anonymously, and each patient was assigned a random numeric code. The collection, transfer, and analysis of data were based on the guidelines for good clinical practice and the relevant national regulations.

### 2.4. Statistical Analysis

Baseline characteristics were summarized using descriptive statistics, including the mean and standard deviation for normally distributed continuous variables, medians and interquartile ranges (IQRs) for non-normally distributed continuous variables, and absolute (N) and relative (%) frequencies for categorical ones. Trends regarding the timing and duration of remdesivir administration were also graphically explored using scatterplots and lowess curves [16].

The time to recovery was analyzed using survival analysis techniques (Kaplan–Meier curves, logrank tests, and multivariable Cox proportional hazards models). For time-to-recovery analyses, data for patients who did not recover and data for patients who died were censored at day 29. All logrank tests and Cox models were stratified by baseline disease severity, which is defined in the same way as in the ACTT-1 clinical trial [9]. More specifically, a severe disease was defined as one that meets one or more of the following criteria: the patient requires invasive or non-invasive mechanical ventilation; supplemental oxygen; SpO2 ≤ 94% room air; a tachypnoea, defined as respiratory rate ≥ 24 breaths per minute. Patients who were admitted to the ICU on the first day were excluded. All other cases were classified as mild/moderate.

Statistical analysis was performed using STATA MP18 (StataCorp. 2023. Stata Statistical Software: Release 18. College Station, TX, USA: StataCorp LLC.).

## 3. Results

### 3.1. Patients’ Characteristics

During the study period (1 September 2020–28 February 2021), 1004 patients were eligible and included in the study. The mean age of the patients was 61.3 (SD 15.0) years, and 60.6% were male. The median number of days between symptom onset and admission was six (IQR, from 4 to 8), irrespective of the disease severity. In total, 746 patients (74.3%) had a severe disease at admission (referred to as the “severe” group), while the rest had a mild/moderate disease (referred to as the “mild/moderate” group). Almost half of the patients (483, 48%) had two or more medical comorbidities (52.01% in the severe group vs. 36.82% in the mild/moderate group, *p* < 0.001). Five hundred twenty-four patients (52.2%) were in need of supplemental oxygen at admission. Among those with deferred administration data, all patients with a severe disease as well as 38/58 (65.5%) patients with a mild/moderate disease started the remdesivir treatment simultaneously with the initiation of supplemental oxygen therapy. Detailed demographic and clinical characteristics of the patients at baseline are presented in Table 1 in terms of disease severity at admission.

### 3.2. Remdesivir Patterns

The median time between admission and remdesivir administration was 1 (IQR, from 1 to 2) day, and the median time between symptoms’ onset and remdesivir administration was 7 (IQR, from 4 to 9) days. Patterns of remdesivir use and other treatments by disease severity at the baseline are presented in Table 2.

The first dose of remdesivir was administered to 871 (86.7%) patients within the first 48 h of their hospital admission (median (IQR) time since symptoms onset being 6 (4–6) days), whereas 133 (13.3%) received remdesivir after the second day of hospitalization (median (IQR) time since symptoms onset being 8 (6–11)). Among the patients in the severe group at admission, almost 90% underwent early remdesivir administration compared to 77.5% in the mild/moderate group (*p* < 0.001). There were 89 (8.9%) people who received a treatment regimen for less than 5 days, 780 (77.7%) who received it for 5 days, and 135 (13.2%) who received it for more than 5 days.

There was a trend for a longer time gap between admission and remdesivir administration during the first 2 months of the study period (Figure S1a), whereas the average duration of remdesivir administration was relatively stable throughout the study period (Figure S1b). No significant associations were found between the timing or duration of remdesivir administration and baseline characteristics described in Table 1.

### 3.3. Effectiveness of Remdesivir

**Figure 2:**
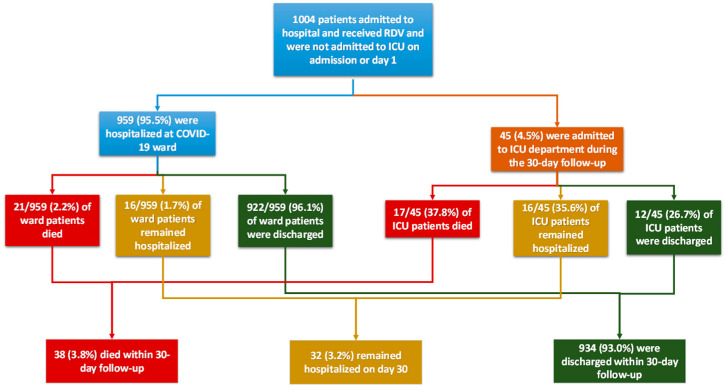
Flow chart describing the clinical progression of the patients within 30 days since admission. Note: All percentages refer to the total study population.

Out of 1004 study patients, 934 (93%) fully recovered (all discharged within 30 days from admission), and 32 (3.2%) patients were still hospitalized 30 days after admission. Thirty-eight (3.8%) patients died in total, with half of them having been previously intubated. In total, 45 (4.5%) patients were admitted to the ICU (Figure 2).

The median time to recovery was 10 (95% CI: 9–10) days. The estimated cumulative probabilities (95% CI) of recovery were 10.9% (9.1–12.9%), 55.5% (52.4–58.6%), 80.9% (78.4–83.2%) and 93% (91.3–94.5%) for the patients 5, 10, 15, and 30 days since admission, respectively.

**Figure 3:**
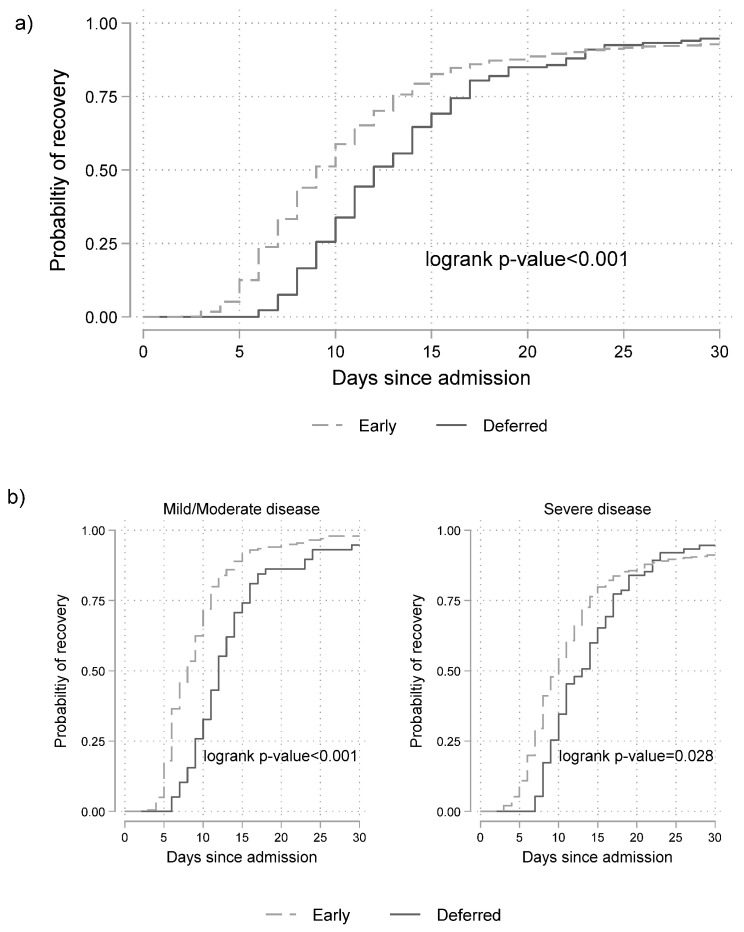
Cumulative probability of recovery since hospital admission (**a**) according to timing of remdesivir administration (logrank test is stratified by baseline disease severity) (“early” (remdesivir initiation in <24 h since hospitalization); “deferred” (remdesivir initiation in ≥24 h since hospitalization)), and (**b**) according to timing of remdesivir administration and disease severity at baseline. Dotted line depicts the “early” group and solid line the “deferred” one.

The cumulative probabilities of recovery since admission according to the timing of remdesivir administration and baseline disease severity are shown in Figure 3.

The median time (95% CI) to recovery was 10 (10–11) days for the severe group and 9 (8–10) days for the mild/moderate group of patients. The cumulative probability of recovery was at all time points higher for the mild/moderate group compared to that of the severe group (*p* < 0.001).

The median time to recovery was 9 (9–10) days for the early and 12 (11–14) days for the deferred remdesivir group (*p* < 0.001).

Stratifying by baseline disease severity, among those with severe disease, the median time (95% CI) to recovery was 10 (9–10) days for the early remdesivir group and 13 (11–15) days (*p* = 0.028) for the deferred remdesivir group. The corresponding figures for those in the mild/moderate group were 8 (7–9) and 12 (11–14) days, respectively (*p* < 0.001); Respective Kaplan–Meier curves are shown in Figure 3b.

In the multivariable analysis, a statistically significant interaction between the timing of remdesivir administration and baseline disease severity (*p* = 0.022) was found. The estimated adjusted HR of recovery for early vs. deferred remdesivir administration was 2.02 (1.49–2.74; *p* < 0.001) among the mild/moderate group patients and 1.27 (1.00–1.63; *p* = 0.054) among the severe group patients. The other factors associated with slower rates of recovery were having an age above 65 years (HR: 0.62, 0.53–0.71; *p* < 0.001) compared to that of younger patients and living in rural areas (HR: 0.74, 0.57–0.96; *p* = 0.023) compared to those living in urban areas with a higher number of coexisting conditions (HR: 0.95 for 1 unit increase, 0.91–1.00; *p* = 0.038) (Table 3). Dexamethasone administration was not statistically significantly associated with the recovery rate in the univariable nor multivariable models, and its inclusion in the multivariable model presented in Table 3 did not alter the effects of remdesivir, as described above.

At 15 days after admission, 48 (4.8%) patients were hospitalized, requiring supplemental oxygen, 17 (1.7%) were on non-invasive ventilation or high-flow oxygen supply, 23 (2.3%) were on invasive mechanical ventilation or extracorporeal membrane oxygenation, and 24 (2.4%) had died (stages 5–8 of the 8-point ordinal scale) (Figure S2). The corresponding figures for the clinical status at 30 days after admission were 2 (0.2%), 5 (0.5%), 8 (0.8%), and 38 (3.8%), respectively. The clinical status according to the 8-point ordinal scale was significantly worse in the patients in the severe group compared to that of the patients in the mild/moderate group both at 15 and 30 days after admission (*p* < 0.001 for both, Figure S3). Similarly, the clinical status according to the 8-point ordinal scale was significantly worse in the deferred group compared to that of the early remdesivir group at 15 days after admission (*p* = 0.001), but there was no significant difference between the two groups at 30 days after admission (*p* = 0.249, Figure S3). The results had a similar trend and a similar statistical significance when stratifying by baseline severity.

The cumulative probabilities of intubation, ICU admission, or death (referred to as composite event) were 6.0% (95% CI: 4.7%–7.6%) and 6.7% (5.3%–8.4%) at 15 and 30 days, respectively (Figure S4a). The cumulative probability of composite events was higher at all the time points for the severe group compared to those of the mild/moderate group (*p* < 0.001), but no noticeable differences between the early and deferred administration groups were observed within the severe group. The stratification by disease severity at admission (Figure S4b) showed that the patients in the mild/moderate group who underwent early remdesivir administration had a lower hazard of progression to the composite event compared to that of the same group of patients, but who underwent deferred remdesivir administration (*p* = 0.044). In the multivariable analysis, a marginally non-significant interaction between the timing of remdesivir administration and baseline disease severity was observed (*p* = 0.053, Table S1); the early administration of remdesivir in patients with a mild/moderate disease was associated with an indicative (HR: 0.20, 0.03–1.23; *p* = 0.083) decrease in the hazard of progression to the composite event, whereas there was no statistically significant difference in the patients with a severe disease (*p* = 0.393). Having an age above 65 years was the only other factor affecting the probability of progressing to a composite event (HR: 2.86, 1.63–5.05; *p* < 0.001) (Supplementary File Table S1).

### 3.4. Safety Analysis

Abnormal liver tests throughout hospitalization were conducted on 130 patients (12.9%). Ninety-three (9.3% of the total cohort) and thirty-seven (3.7% of the total cohort) patients had stage 1 and stage 2 liver injuries, respectively. Remdesivir treatment was stopped due to abnormal liver tests in four (0.4%) cases. Sixty-one out of one hundred and thirty (46.9%) patients with abnormal liver results were discovered to have them before remdesivir administration (6.1% of total cohort), and another 61/130 (46.9%) were found during the remdesivir treatment or up to two days after the end of administration (6.1% of the total cohort), and 8/130 (6.2%) were found after this period (0.8% of the total cohort).

An acute kidney injury during the hospitalization period was recorded in 30 (3.0%) patients. Type-I AKI was detected in 25 (2.5%) patients, type-II was detected in 5 (0.5%) patients, and nobody experienced type-III AKI. All the patients who experienced type-II AKI had severe COVID-19 at admission. One patient had to discontinue remdesivir due to AKI. AKI occurred in 9/30 (30%) patients before the initiation of remdesivir (0.9% of total cohort), 16/30 (53.3%) had it during remdesivir administration or up to 2 days after the end of administration (1.6% of the total cohort), and 5/30 (16.7%) patients had it after this period (0.5% of the total cohort). Two (0.2%) patients were classified as type-I AKI before remdesivir initiation and progressed to type-II AKI during the treatment.

Nine (0.9%) patients interrupted the remdesivir treatment because of the side effects.

## 4. Discussion

In this multicenter retrospective observational study from six hospitals during the second wave of the pandemic in Greece (September 2020–February 2021), we described the patterns of use, effectiveness, and safety of remdesivir among hospitalized patients with COVID-19. Most of the patients (78%) received a 5-day scheme, as recommended by the national guidelines at that time, and 87% initiated remdesivir within the first two days of their hospitalization. The administration of remdesivir within the first 48 h after admission was associated with a faster recovery, with the median times to hospital discharge being 4 and 3 days shorter among those with mild/moderate and severe diseases, respectively. The majority (93%) of the patients were discharged, while 38 (3.8%) died within 30 days after admission. Only nine (0.9%) patients had to stop their remdesivir treatment because of adverse events.

Following its first conditional approval by the EMA in July 2020, Greece included remdesivir as a formal treatment recommendation for patients with severe COVID-19 disease in the national COVID-19 treatment algorithm. Remdesivir was the only recommended antiviral to be used either alone or in combination with dexamethasone and anticoagulants according to the clinical status, which is in accordance with other international guidelines [17]. The majority of hospitalized patients in the study were treated with remdesivir in accordance with national recommendations for five days. The second wave of the pandemic in Greece was particularly severe and posed a great burden on the NHS. The high patient numbers during the peak of this wave may have led physicians to terminate remdesivir treatments for ready-to-discharge patients earlier than 5 days, resulting in schemes that lasted for 4 days or less for 8.9% of the study participants. At this point, it should be noted that remdesivir was approved only for the treatment of a severe disease for a minimum of 5 days to a maximum of 10 days, and no label indication existed for the 3-day regimen during the study period. This came later, in December 2021, after the publication of the PINETREE study results [18].

ReEs-COVID-19 confirms the pattern of early remdesivir administration almost concurrently with or right away after admission. This is consistent with the concept of the maximal effectiveness of antivirals in relation to the timing of their administration, which is also evident in clinical trials such as ACTT-1 and real-world studies [19], with it becoming the rule after October 2020. Logistic hurdles mainly related to the requirement for a pre-approval process for the prescription by the national authority until October 2020 contributed to the relatively longer delays in remdesivir administration in September-October 2020. A similar trend was also described in the USA where the rate of remdesivir use increased from 39.9% in October to 52.2% in December 2020 [20].

Our results are consistent with those from the ACTT-1 randomized clinical trial, with the median time to recovery being exactly the same (10 days). However, the 30-day mortality rate (3.8%) was lower than those in the ACTT-1 (11%), WHO’s Solidarity (15%), and DisCoVeRy (8%) trials [9,12,21]. In a multicenter international study using data from both an open-label trial and a real-world cohort, the rates of recovery at 14 days since admission were slightly lower (74.4%) compared to those observed in our study (80.9%) [22]. In most [23,24,25], but not all [26] the observational studies, the 30-day mortality and/or disease progression rates among patients treated with remdesivir for 5 days were higher than those in our study, at around 12–13%. In a recently published Greek study, a 10% mortality rate was reported in three Greek hospitals (4% in 14 days), with a 9-day mean interval between admission and recovery [27]. The hospitals participating in our study are major reference centers with significant clinical and research experience and are on the frontlines of COVID-19 clinical trials (national or international randomized clinical trials, RCTs). During the study period, several RCTs on hospitalized COVID-19 patients were in effect; based on personal communication with study physicians, there was a tendency for patients with a more severe COVID-19 disease to be preferentially enrolled in RCTs, thus rendering them non-eligible for our study. On one hand, this may partially explain the significantly lower fatality rate among ReEs-COVID-19 patients compared to those in similar studies; other differences, e.g., in the patients’ risk factors and/or comorbidities, may also have contributed to the observed difference in the mortality rates. On the other hand, the majority of the patients enrolled in this study belonged to the low-flow oxygen group, in which a mortality benefit due to the use of remdesivir was recorded in many RCTs, including ACTT-1 and Solidarity, plus real-world studies [9,12].

During the study period, the in-patient mortality in Greece increased, particularly among COVID-19 patients [27]. In a single-center study in Greece by Cholongitas et al. with 1046 COVID-19 patients for the period March 2020-October 2021, via multivariable analysis, the authors found that remdesivir administration was a protective factor against mortality (HR 0.50, 95%CI 0.30–0.85; *p* = 0.009) [28].

Remdesivir administered within 24 h of admission further reduced the length of hospital stays by 4 days for those that presented with a mild/moderate disease and by 3 days for those that presented with a severe disease compared to those who received it later, freeing up beds for incoming patients. Early remdesivir administration may have contributed to improving the resilience of healthcare systems during the hard times of the early COVID-19 epidemic. ReEs-COVID-19 identified the same covariates for poor hospitalization outcomes among COVID-19 patients (age over 65 and rural domicile) as those previously published for the same research period, but also highlighted the significance of pre-existing comorbidities [29]. Having a non-Greek origin was not identified as a prognostic factor in our study; other studies have reported that having a non-dominant ethnic origin and/or deprivation are associated with the increased risk of disease progression and mortality [30,31,32]. In our study, though, the proportion of those of non-Greek origin was relatively small, so the power to detect significant differences was low.

ReEs-COVID-19 reinforces the established safety profile of remdesivir [33,34]. A relatively small number of patients (n = 69; 6.9%) had abnormal liver function test results after remdesivir initiation, and only four of them had to prematurely discontinue remdesivir. In two other studies, patients receiving remdesivir experienced fewer such abnormalities than those receiving placebo did [33,34]. Liver hepatotoxicity in ReEs-COVID-19 patients may also be in part caused by the SARS-CoV-2 hepatotoxic profile and not solely due to a remdesivir-induced liver injury [35]. This assumption is supported by the fact that 6.1% of ReEs-COVID-19 patients presented with abnormal liver function test results before remdesivir administration. In the study by Cholongitas et al. [28], at admission, 34.7% and 25.7% patients had abnormal AST and ALT values, respectively, while during hospitalization, 53 (5%) patients fulfilled the criteria for a liver injury, where remdesivir was not identified as a risk factor. Interestingly, in this study, patients with abnormal baseline AST values (i.e., >40 IU/L) had worse outcomes compared to those with normal AST values (log rank test: chi square: 8.8, *p* = 0.003) [28].

AKI was observed in nine (0.9%) and twenty-one (2.1%) ReEs-COVID-19 patients before and after remdesivir administration, and only one had to prematurely discontinue remdesivir. The poor prognosis for COVID-19 patients with AKI complications has already been described [36,37]. Even if AKI is uncommon in COVID-19 patients receiving remdesivir, there should be appropriate renal function monitoring and a pharmacovigilance alert [38,39,40,41]. COVID-19 can result in renal impairment; however, its prompt treatment may contribute to preventing AKI occurrence [42,43]. In the study of Lim et al., remdesivir initiation was negatively associated with AKI events (OR: 0.40; 95% CI: 0.24–0.67, *p* ≤ 0.001). In addition, the ACTT-1 study did not report any differences concerning AKI events between the remdesivir and placebo groups, and the PINETREE study reported a minimal change from the baseline in creatinine clearance in the remdesivir group [9,18,32]. In any case, the assessment of patients with COVID-19 renal function should be monitored before and during the treatment with remdesivir.

The limitations of the current study include its observational, retrospective, and single-arm nature, which rendered comparisons with other plausible treatments irrelevant. Additionally, as our data reflect real clinical practice, patients with a severe disease at admission were more likely to initiate remdesivir sooner. Moreover, during the ReEs-COVID-19 period, many clinical trials were being conducted in Greek hospitals; hence, many COVID-19 hospitalized patients were ineligible for inclusion in this cohort study. Finally, the ReEs-COVID-19 period coincides with the dominance of the Delta variant, making it crucial to exercise caution when interpreting the study’s results due to the possible presence of other variants with a modified pathogenicity. It is noteworthy, though, that in a more recent Greek study of patients with SARS-CoV-2 pneumonia (69% with the Omicron-1 variant and 31% with the Delta variant), remdesivir was associated with reduced mortality, while the recovery rates did not differ significantly by variant [44].

Despite the aforementioned limitations, our study reflects the experience of remdesivir in real life during a time of increased hospital bed demand during the second wave of the pre-vaccination era. Our data include clinical experience both in large centers in the capital and in centers covering the healthcare needs of patients residing in semi-urban and rural regions of the country.

## 5. Conclusions

During the second wave of the pandemic in Greece, remdesivir was used by patients with a need for oxygen supplementation in a 5-day regimen. ReEs-COVID-19 confirmed the increased efficacy of remdesivir when administered within the first 24 h after hospital admission, and it had a safe and predictable profile.

## Figures and Tables

**Table 1:** Patients’ characteristics, coexisting conditions and 8-degree ordinal scale status by disease severity at baseline (i.e., hospital admission).

Disease Severity at Baseline *				
	Severe *n* = 746 (74.30%)	Mild/Moderate *n* = 258 (25.70%)	Overall *n* = 1004 (100%)	*p*-Value
Age (years)—Mean (SD)	62.6 (14.6)	57.8 (15.6)	61.3 (15.0)	<0.001
Male	454 (60.86%)	154 (59.69%)	608 (60.56%)	0.768
Greek origin	685 (91.82%)	233 (90.31%)	918 (91.43%)	0.437
Residence				0.152
*Rural*	60 (8.04%)	14 (5.43%)	74 (7.37%)	
*Urban*	618 (82.84%)	212 (82.17%)	830 (82.67%)	
*Semi-urban*	68 (9.12%)	32 (12.40%)	100 (9.96%)	
Smoking				0.001
*Active Smoker*	49 (6.57%)	19 (7.36%)	68 (6.77%)	
*Never Smoker*	303 (40.62%)	152 (58.91%)	455 (45.32%)	
*Former Smoker*	140 (18.77%)	32 (12.40%)	172 (17.13%)	
Alcohol abuse	7 (0.94%)	2 (0.78%)	9 (0.90%)	>0.999
Region				0.079
*Attica*	417 (55.90%)	161 (62.40%)	578 (57.57%)	
*Rest of Greece*	329 (44.10%)	97 (37.60%)	426 (42.43%)	
Days from symptoms onset to admission—Median (IQR) Comorbidities	6.0 (4.0, 8.0)	6.0 (4.0, 8.0)	6.0 (4.0, 8.0)	0.282
COPD	55 (7.37%)	7 (2.71%)	62 (6.18%)	0.006
Diabetes mellitus	188 (25.20%)	48 (18.60%)	236 (23.51%)	0.033
Obesity	105 (14.08%)	27 (10.47%)	132 (13.15%)	0.164
CVD	70 (9.38%)	31 (12.02%)	101 (10.06%)	0.231
Number of comorbidities				<0.001
*None*	192 (25.74%)	100 (38.76%)	292 (29.08%)	
*One*	166 (22.25%)	63 (24.42%)	229 (22.81%)	
*Two or more*	388 (52.01%)	95 (36.82%)	483 (48.11%)	
Baseline severity				<0.001
*Hospitalized, not requiring supplemental oxygen, but requiring ongoing medical care*	136 (18.23%)	258 (100.00%)	394 (39.24%)	
*Hospitalized, requiring supplemental oxygen*	524 (70.24%)	0 (0.00%)	524 (52.19%)	
*Hospitalized, on non-invasive ventilation or high flow oxygen devices*	86 (11.53%)	0 (0.00%)	86 (8.57%)	

* A severe disease was defined as meeting one or more of the following criteria: the patient requires invasive or non-invasive mechanical ventilation, supplemental oxygen, SpO2 ≤ 94% room air, or a tachypnoea defined as respiratory rate ≥ 24 breaths per minute. All other cases were classified as mild/moderate disease.

**Table 2:** Patterns of remdesivir use and other treatments by disease severity at baseline.

	Disease Severity at Baseline			
	Severe n = 746 (74.30%)	Mild/Moderate n = 258 (25.70%)	Overall n = 1004 (100%)	
Remdesivir administration				<0.001
*Deferred Administration*	75 (10.05%) *	58 (22.48%) *	133 (13.25%)	
*Early Administration*	671 (89.95%)	200 (77.52%)	871 (86.75%)	
Duration of remdesivir administration (categories)				0.674
*<5 days*	70 (9.38%)	19 (7.36%)	89 (8.86%)	
*5 days*	575 (77.08%)	205 (79.46%)	780 (77.69%)	
*6 to 10 days*	98 (13.14%)	34 (13.18%)	132 (13.15%)	
*>10 days*	3 (0.40%)	0 (0.00%)	3 (0.30%)	
Duration of remdesevir administration (days)—*Median* (*IQR*) (*min-max*)	5.0 (5.0, 5.0) (1.0, 12.0)	5.0 (5.0, 5.0) (1.0, 10.0)	5.0 (5.0, 5.0) (1.0, 12.0)	0.713
Time between symptoms onset and remdesivir administration (days)—*Median* (*IQR*) (*min*,*max*)	7 (4, 9) (0, 18)	7 (5, 9) (0, 21)	7 (4, 9) (0, 21)	0.201
Days from admission to Remdesivir administration—*Median* (*IQR*) (*min*,*max*)	1 (1, 2) (1, 13)	1 (1, 2) (1, 12)	1 (1, 2) (1, 13)	<0.001
Dexamethasone **	646 (86.60%)	132 (51.16%)	778 (77.49%)	<0.001
Anticoagulants	723 (96.92%)	248 (96.12%)	971 (96.71%)	0.545

* Among those with deferred administration, all patients with severe disease as well as 38/58 (65.5%) patients with mild/moderate disease started remdesivir treatment simultaneously with the initiation of supplemental oxygen therapy; ** 759/778 patients who received dexamethasone started this treatment at the time they needed supplemental oxygen therapy.

**Table 3:** Results from the final multivariable Cox proportional hazards model for the probability of recovery according to timing of remdesivir administration. Model was stratified by disease severity at baseline.

	Hazard Ratio (HR)	95% C.I.	*p*-Value
Remdesivir administration within first 2 days/after 2nd day since admission (mild/moderate disease at baseline)	2.02	(1.49, 2.74)	<0.001
Remdesivir administration within 2 days/after 2nd day since admission (severe disease at baseline)	1.27	(1.00, 1.63)	0.054
Interaction: Disease severity X timing of Remdesivir			0.022
Number of co-existing conditions	0.95	(0.91, 1.00)	0.038
Age above or equal to 65/below 65 years	0.62	(0.53, 0.71)	<0.001
Residence			
*Rural*/Urban	0.74	(0.57, 0.96)	0.023
*Semi-urban*/Urban	0.98	(0.79, 1.22)	0.850

## Data Availability

ReEs-COVID 19 data are derived from collaborating clinics, and although individual data do not include patient names or identifying information of the participants, as data contain potentially sensitive information, there are ethical restrictions imposed by the Bioethics and Deontology Committee of the Medical School of the National and Kapodistrian University of Athens. Anonymized individual data can be shared after interested researchers submit a concept sheet to the AMACS steering committee (chair: Giota Touloumi; email: gtouloum@med.uoa.gr) and the Bioethics and Deontology Committee of the Medical School of the National and Kapodistrian University of Athens (bioethics@med.uoa.gr).

## Supplementary Materials

The following supporting information can be downloaded at: https://www.mdpi.com/article/10.3390/microorganisms11081998/s1, Table S1. Results from the final multivariable Cox proportional hazards model for the hazard of experiencing the composite event (intubation, ICU admission, or death) according to timing of remdesivir administration stratified according to disease severity at baseline; Figure S1a. Time from admission to remdesevir administration according to calendar time of admission; Figure S1b. Duration of remdesivir administration according to calendar time of admission; Figure S2. Clinical status (8-degree ordinal scale) at baseline and days 15 and 30; Figure S3. Clinical status (8-degree ordinal scale) at baseline and days 15 and 30 according to disease severity at baseline and timing of remdesivir administration; Figure S4a. Cumulative probability of composite event according to time since admission and disease severity at baseline; Figure S4b. Cumulative probability of composite event according to time since admission and timing of remdesivir administration stratified according to disease severity at baseline.
