# Supplementary material for: Remdesivir: Effectiveness and Safety in Hospitalized Patients with COVID-19 (ReEs-COVID-19)—Analysis of Data from Daily Practice
Source: Microorganisms. 2023 Aug 3;11(8):1998. doi: 10.3390/microorganisms11081998 (PMC10459397; doi:10.3390/microorganisms11081998)

Supplemental Tables and Figures:

Table S1. Results from the final multivariable Cox proportional hazards model for the hazard of experiencing the composite event (intubation, ICU admission or death) by timing of Remdesivir administration. Stratified by disease severity at baseline.

Covariate	Hazard Ratio (HR)	95% C.I.	p-value
Early Remdesivir administration (mild/moderate disease at baseline)	0.20	(0.03, 1.23)	0.083
Deferred Remdesivir administration (severe disease at baseline)	1.49	(0.60, 3.74)	0.393
Interaction: Disease severity X timing of Remdesivir			0.053
No. of coexisting conditions	1.15	(0.99, 1.35)	0.072
Age above or equal to 65 vs. below 65	2.86	(1.63, 5.05)	<0.001

Figure S1a. Time from admission to Remdesevir administration by calendar time of admission

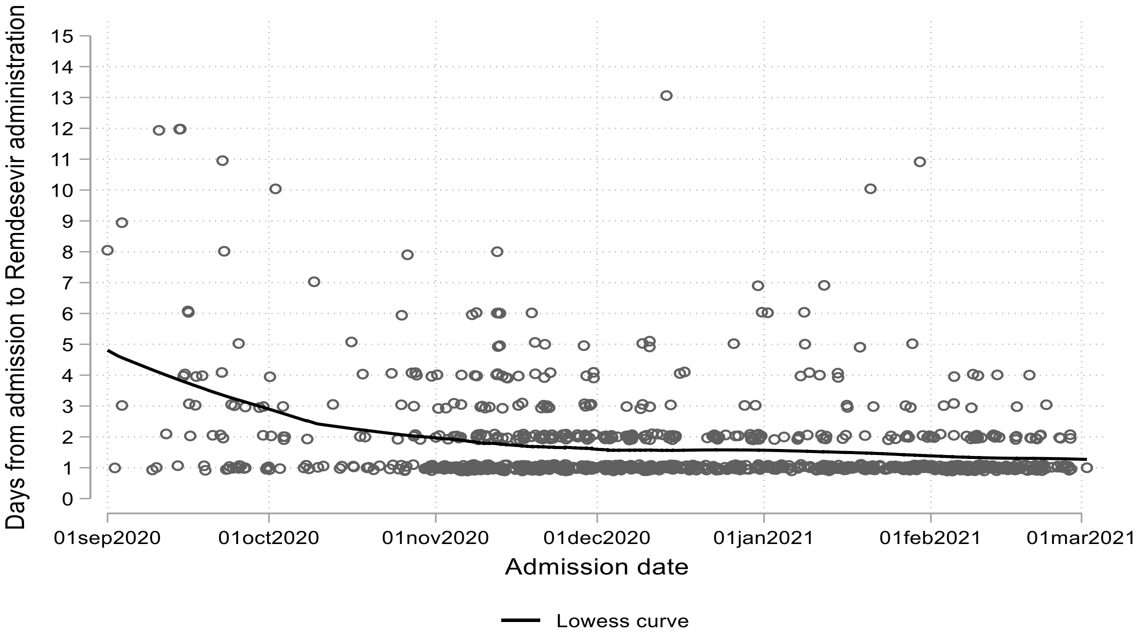

Figure S1b. Duration of Remdesivir administration by calendar time of admission

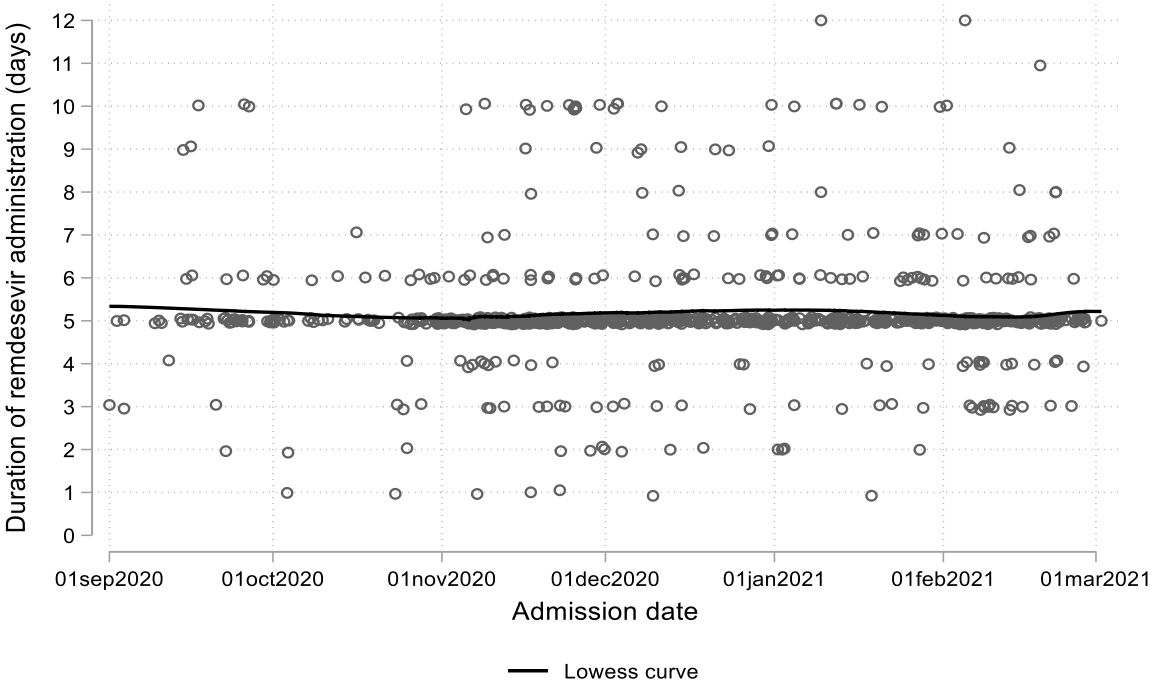

Figure S2 . Clinical status (8-degree ordinal scale) at baseline and days 15 and 30.

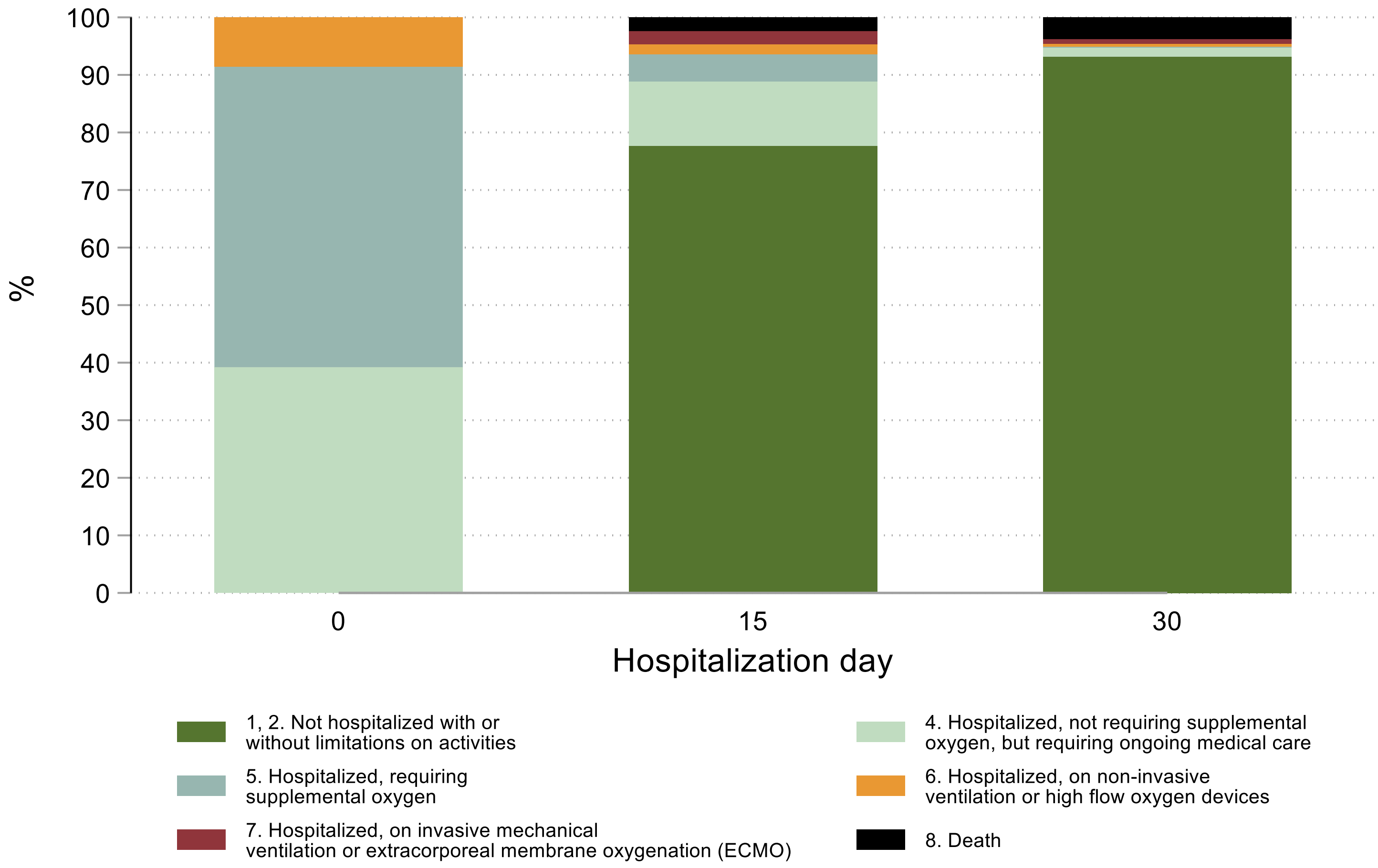

Figure S3 . Clinical status (8-degree ordinal scale) at baseline and days 15 and 30 by disease severity at baseline and timing of remdesivir administration.

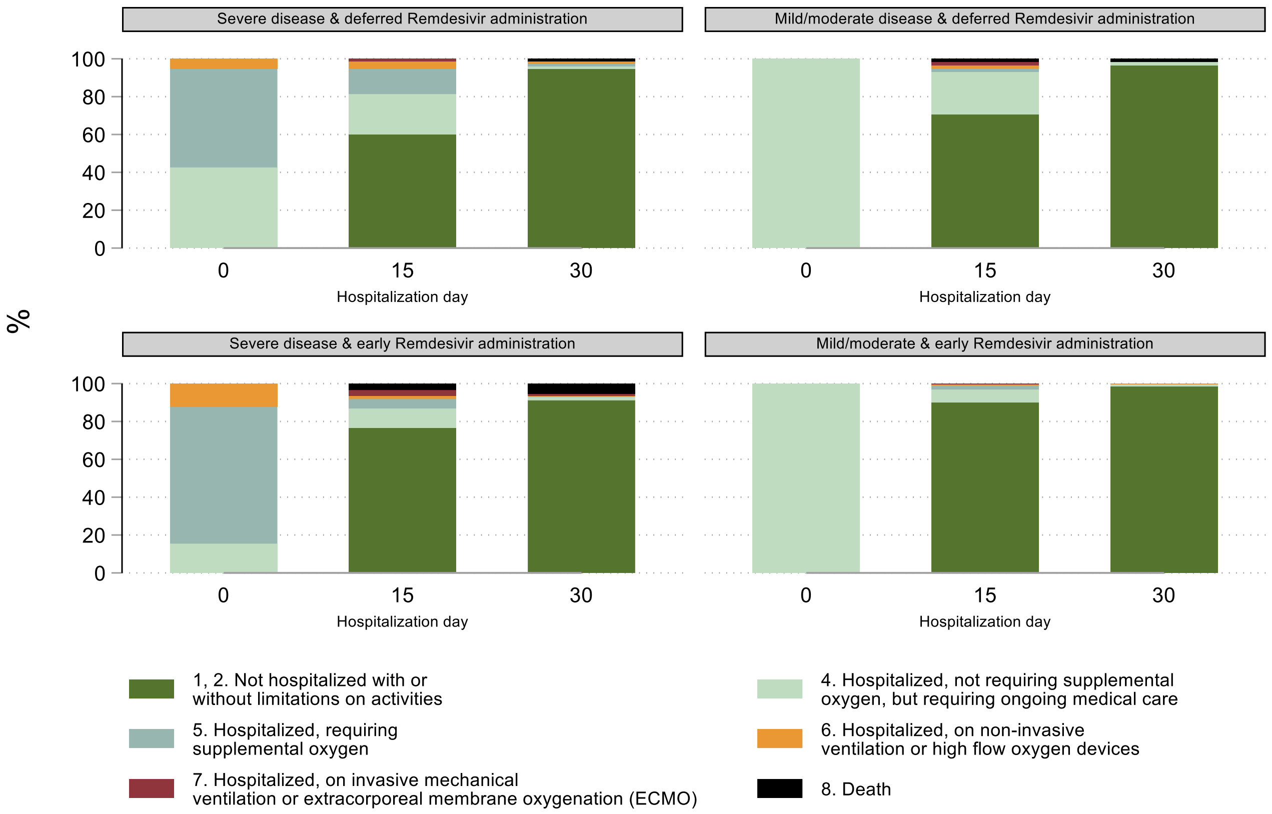

Figure S4a. Cumulative probability of composite event by time since admission and disease severity at baseline.

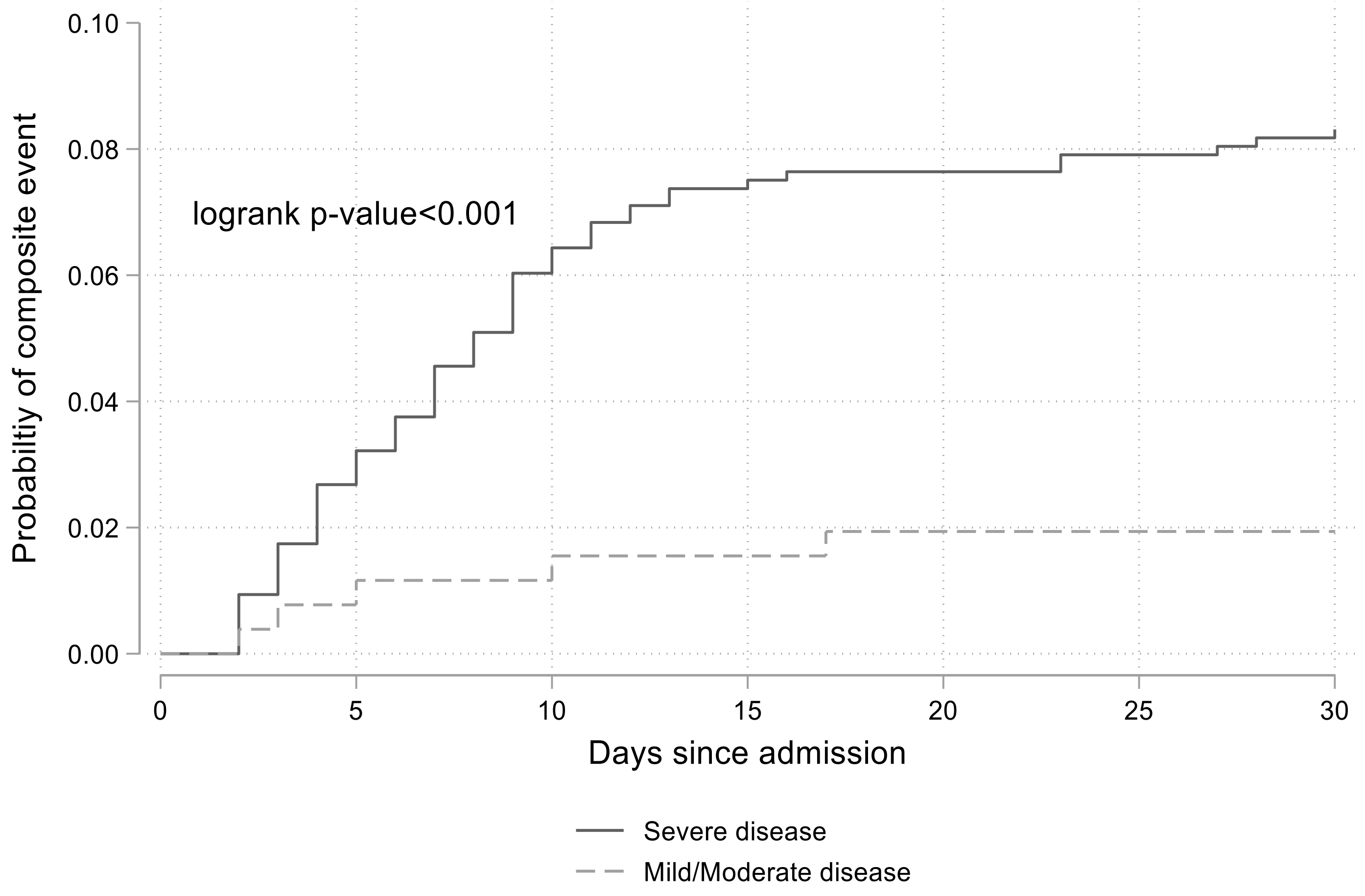

Figure S4b. Cumulative probability of composite event by time since admission and timing of Remdesivir administration stratified by disease severity at baseline.

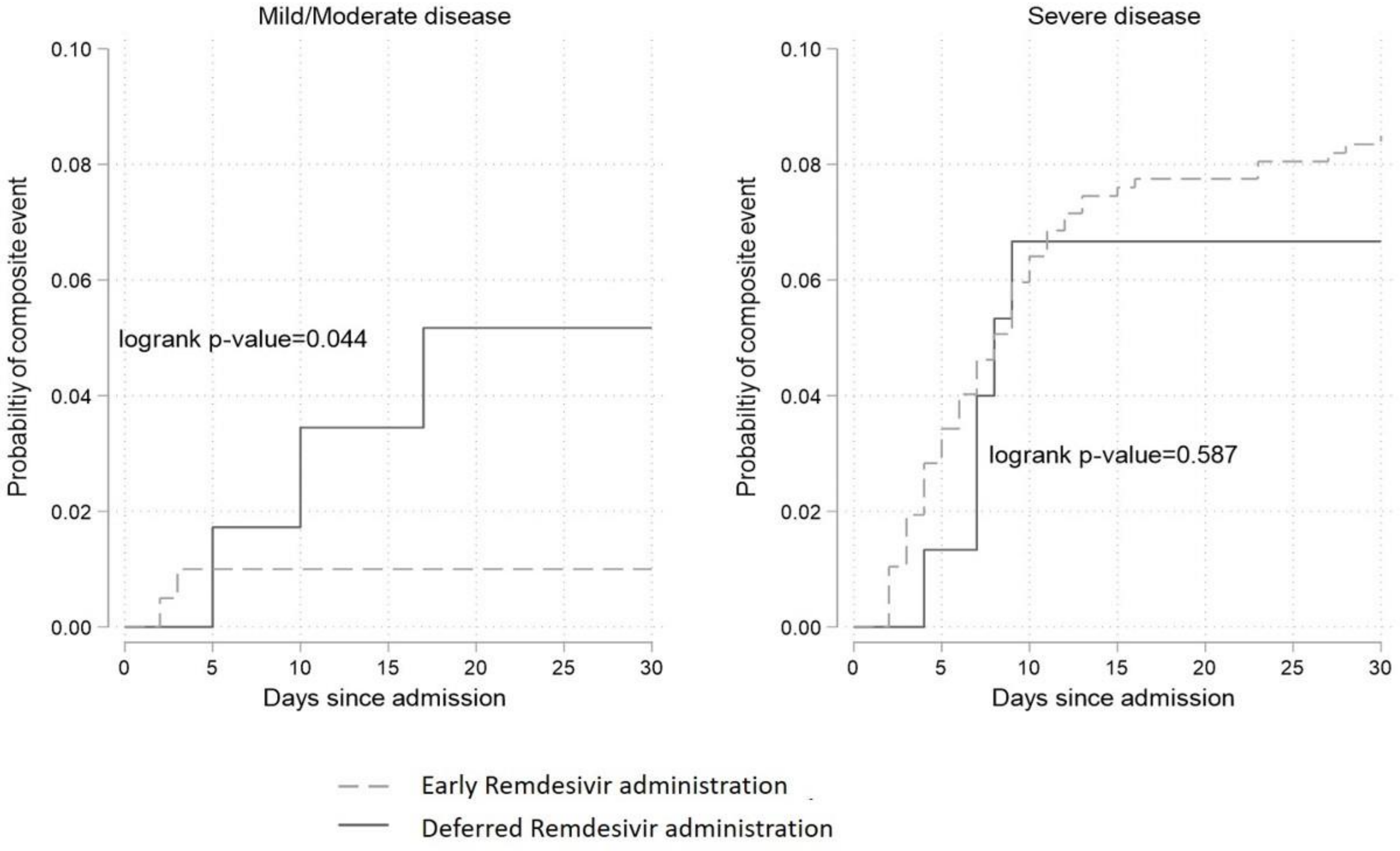

Supplement: Supplementary file 1 [file microorganisms-11-01998-s001.zip › microorganisms-2515431-supplementary.pdf]
